# The Interplay of the Unfolded Protein Response in Neurodegenerative Diseases: A Therapeutic Role of Curcumin

**DOI:** 10.3389/fnagi.2021.767493

**Published:** 2021-11-19

**Authors:** Sitabja Mukherjee, Awdhesh Kumar Mishra, G. D. Ghouse Peer, Sali Abubaker Bagabir, Shafiul Haque, Ramendra Pati Pandey, V. Samuel Raj, Neeraj Jain, Atul Pandey, Santosh Kumar Kar

**Affiliations:** ^1^School of Biotechnology, KIIT University, Bhubaneswar, India; ^2^Department of Biotechnology, Yeungnam University, Gyeongsan, South Korea; ^3^Department of Biotechnology, Sri Ramaswamy Memorial (SRM) University, Sonepat, India; ^4^Department of Medical Laboratory Technology, Faculty of Applied Medical Sciences, Jazan University, Jazan, Saudi Arabia; ^5^Research and Scientific Studies Unit, College of Nursing and Allied Health Sciences, Jazan University, Jazan, Saudi Arabia; ^6^Faculty of Medicine, Bursa Uludağ University, Bursa, Turkey; ^7^Division of Cancer Biology, Council of Scientific and Industrial Research (CSIR)-Central Drug Research Institute, Lucknow, India; ^8^Department of Ecology and Evolutionary Biology, University of Michigan, Ann Arbor, MI, United States; ^9^Department of Ecology, Evolution and Behavior, The Alexander Silberman Institute of Life Science, The Hebrew University of Jerusalem, Jerusalem, Israel; ^10^Nano Herb Research Laboratory, Kalinga Institute of Industrial Technology (KIIT) Technology Business Incubator, KIIT University, Bhubaneswar, India

**Keywords:** Alzheimer’s disease, Parkinson’s disease, neurodegenaration, unfolded protein response, ER stress, curcumin, ROS—reactive oxygen species, cell death

## Abstract

Abnormal accumulation of misfolded proteins in the endoplasmic reticulum and their aggregation causes inflammation and endoplasmic reticulum stress. This promotes accumulation of toxic proteins in the body tissues especially brain leading to manifestation of neurodegenerative diseases. The studies suggest that deregulation of proteostasis, particularly aberrant unfolded protein response (UPR) signaling, may be a common morbific process in the development of neurodegeneration. Curcumin, the mixture of low molecular weight polyphenolic compounds from turmeric, *Curcuma longa* has shown promising response to prevents many diseases including current global severe acute respiratory syndrome coronavirus 2 (SARS-CoV-2) infection and neurodegenerative disorders. The UPR which correlates positively with neurodegenerative disorders were found affected by curcumin. In this review, we examine the evidence from many model systems illustrating how curcumin interacts with UPR and slows down the development of various neurodegenerative disorders (ND), e.g., Alzheimer’s and Parkinson’s diseases. The recent global increase in ND patients indicates that researchers and practitioners will need to develop a new pharmacological drug or treatment to manage and cure these neurodegenerative diseases.

## Introduction

The global burden of neurological diseases are rising, and considered as one of the leading causes of mortality and disability across the globe (Gammon, 2014; Feigin et al., 2019). The correct folding and packaging of the proteins are essential in regulation of many neurological diseases. All proteins bound to organelles and extracellular spaces are subject to proteostasis (Wang and Kaufman, 2016).

**FIGURE 1:**
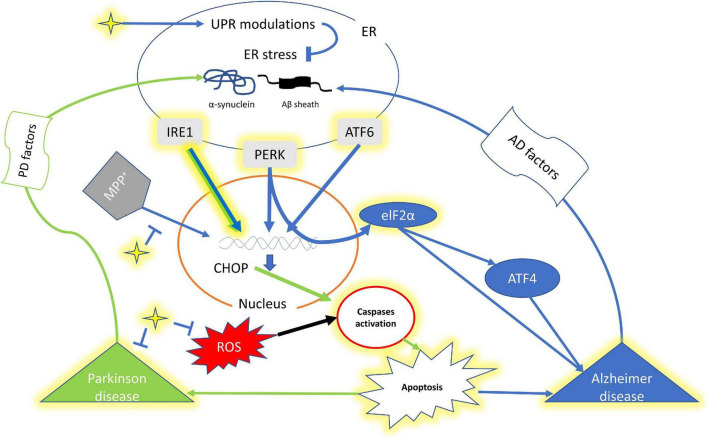
A schematic representation of interplay of curcumin, UPR and factors of Alzheimer’s disease and Parkinson’s disease. The yellow highlighted genes, component and pathway represents the effect of Curcumin. The star shape (yellow) denotes curcumin. Green arrow shows signaling pathway for PD while Blue of AD. ER, endoplasmic reticulum; IRE1, inositol requiring enzymes 1; PERK, protein kinase R-like ER kinase; ATF4, Activating transcription factor 4; ATF6, Activating transcription factor 6; CHOP, C/EBP Homologous Protein; elF2a, Eukaryotic translation initiation factor 2 alpha.

The abundance of too many secretory proteins in the endoplasmic reticulum (ER) induces the unfolded protein response (UPR) and in case of chronic ER-stress this leads to apoptotic cell death (Taniguchi and Yoshida, 2011; Hetz et al., 2013; Shah et al., 2015; Figure 1). Thus, the UPR protects cells against deformed proteins and maintains cellular homeostasis (Smith and Mallucci, 2016). There are three signal transducers inside the ER, which are inositol requiring enzymes 1 (IRE1) α and β, protein kinase R-like ER kinase (PERK) categorized as type I, and activating transcription factor 6 (ATF6), α and β as type II (Shah et al., 2015; Hetz and Papa, 2018). Various transcription factors activate these signal transducers to restore proteostasis and enhance ER and Golgi biogenesis (Hetz et al., 2020). Neurodegenerative diseases (NDs) are characterized by the degeneration and death of neurons (Pohl et al., 2018). While, the misfolded proteins cause ER-stress-induced neuronal apoptosis in progressive neurodegenerative diseases like Alzheimer’s disease (AD), Parkinson’s disease (PD) (Hetz and Mollereau, 2014; Scheper and Hoozemans, 2015; Figure 1). AD which accounts for 60–80% and other forms of dementia are the world’s fifth leading cause of death, and its prevalence is expected to triple by 2050, according to WHO (Voulgaropoulou et al., 2019). While, PD is the most common neurodegenerative disease (20–30%) after Alzheimer’s, with a prevalence of 150/100,000 (Schapira, 1999).

Curcumin, a primary natural polyphenol derived from *Curcuma longa* rhizome is found affecting a number of diseases including current SARS-CoV-2 infection (Gupta et al., 2013; Noorafshan and Ashkani-Esfahani, 2013; Marton et al., 2020; Sharifi-Rad et al., 2020; Ahmadi et al., 2021). The antioxidant, anti-inflammatory, anti-mutagenic, anti-parasitic, antimicrobial and anti-cancerous properties of curcumin are quite explored (Reddy et al., 2005; Aggarwal and Harikumar, 2009; Gupta et al., 2013; Vera-Ramirez et al., 2013; Lestari and Indrayanto, 2014; Hewlings and Kalman, 2017). Recently curcumin was characterized as a pharmacophore by X-ray micro-crystallography of fiber-forming tau fragments with small molecule binders, binding to the β-pleated layer in tau’s paired helical filaments (Landau et al., 2011). Reports suggest that curcumin further scavenges the toxic reactive oxygen species (ROS) and increases Superoxide dismutase (SOD), Na^+^-K^+^ ATPase, catalase, glutathione, and mitochondrial complex enzyme levels (Reeta et al., 2010; Barzegar and Moosavi-Movahedi, 2011; Li et al., 2020). It also reduces lipid peroxidation by reducing Malondialdehyde, nitrite, and acetylcholinesterase (Abdel-Diam et al., 2019; Kheirouri and Alizadeh, 2019). Curcumin affects neurogenesis in brain regions involving the Canonical Wnt/-Catenin Pathway (Tiwari et al., 2014). It activates nuclear factor erythroid 2-related factor (Nrf2), the antioxidant master regulator, to protect dopaminergic neurons (Dong et al., 2018). Furthermore, curcumin increases the expression of β-tubulin, neuroD1, doublecortin, neurogenic, neuroligin, and neuregulin while decreases the expression of the signal transducer and activator of transcription 3 (STAT3) (Karlstetter et al., 2011). Curcumin further helps in inhibition of neuroinflammation possibly by binding to the sensile plaques, inhibiting Aβ plaque aggregation, plaque pathology, and decreasing amyloid levels (Lim et al., 2001; McClure et al., 2017; Mei et al., 2020). It also inhibits several dysregulated cell-signaling pathways (Su et al., 2020; Ege, 2021). Curcumin also safeguards against AD (Desai and Patravale, 2018; Nebrisi et al., 2020). Recently, curcumin and curcuminoids were reported as a promising candidate against NDs (Gregory et al., 2021; Ryskalin et al., 2021; Silvestro et al., 2021; Simeonova et al., 2021). Here we review the curcumin’s potential role in NDs and UPR regulations. Given the volume of literature, we chosen to focus on with common NDs like AD and PD in relation to UPR.

## Unfolded Protein Response and Neurodegenerative Diseases: A Common Connection and Consequences

UPR activation has been observed in many NDs (Scheper and Hoozemans, 2015). Activated astrogliosis, brain aggravation, and microglial multiplication cause ADs (Haass and Selkoe, 2007). The amyloid-β (Aβ) peptide aggregates in specific brain areas like the neocortex, hippocampus, and limbic region which further causes synaptic failure and neuronal death (Chyung et al., 2005). UPR is a cellular stress reaction caused by misfolded proteins in the ER, while misfolded proteins accumulate in PD (Taylor et al., 2002). Thus, the UPR is linked to PD in cell models (Hoozemans et al., 2007). In PD, Lewy bodies and protein incorporation in neurites were increased (Mahul-Mellier et al., 2020). Synuclein, a small presynaptic protein, is a major component of Lewy bodies (Clayton and George, 1999; Breydo et al., 2012). The PD patients were also found correlated with higher levels of pPERK, peIF2, and pIRE1 than non-neurological controls (Hoozemans et al., 2007). pPERK-positive neurons had increased diffuse cytoplasmic synuclein immunoreactivity. These findings suggest a link between synuclein accumulation and ER stress in dopaminergic neurons. Further, heat shock reactions and ER or mitochondrial unfolded protein reactions are examples of misfold UPR (Lee et al., 2011; Kakkar et al., 2014; Chiti and Dobson, 2017; Shamsi et al., 2017). By upregulating atomic chaperones and proteasome components, the authors reported the increase in the ability to unfold and refold misfolded proteins, as well as eliminate misfolded proteins (Ciechanover and Kwon, 2017).

## Curcumin and Neurodegenerative Diseases: Interventions and Modalities

This section reviews curcumin’s use and effects on NDs, while focusing on PD and AD. Curcumin’s neurological effects make it one of the most promising natural therapies for AD (Noorafshan and Ashkani-Esfahani, 2013; Eghbaliferiz et al., 2020). The lower prevalence of AD in India among adults aged 70–79 years (4.4 times lower than in the US) is attributed to higher curcumin use (Ganguli et al., 2000; Yang et al., 2005). Curcumin’s ability to bind to Aβ-pleated structure reduces plaque stress in most AD plaque pathogenesis models (Yang et al., 2005; Garcia-Alloza et al., 2007; Cheng et al., 2013). Curcumin is also known for directly binding and inhibiting the aggregation of Aβ-sheet conformations found in many NDs (Cole et al., 2007; Mishra and Palanivelu, 2008; Forouzanfar et al., 2020; Radbakhsh et al., 2021).

Curcumin inhibits tau aggregation by binding to neurofibrillary tangles (Brunden et al., 2010; Mohorko et al., 2010; Mutsuga et al., 2012). Several β-pleated layer complexes, such as huntingtin, prion aggregates and α-synuclein, are found interacting with curcumin (Caughey et al., 2003; Ono et al., 2004; Pandey et al., 2008). Curcumin interacts directly with heat shock proteins (HSPs), such as HSP90 and HSP70, in Aβ-infused rats, tau transgenic mice and human models (Ma et al., 2013). It affects phagocytic cell association with plaque structures and stimulates clearance of Aβ aggregates in human cell lines and rodent AD models, similar to the amyloid vaccine (Frautschy et al., 2001; Cole et al., 2004). Curcumin also inhibits NF-κ *B* and Activator Protein 1 (AP1). A dysfunctional transcription factor pathway limits the resolution of inflammation in AD. Curcumin’s inhibition of AP1 transcription results in hyperphosphorylation of tau (Singh and Aggarwal, 1995; Xu et al., 1997; Cho et al., 2007). According to recent research, curcumin seems to decrease peroxisome proliferator activated receptor (PPAR) activation by inhibiting Toll-like receptor 4 complex homodimerization. Wang et al. (2010) report that curcumin directly increases PPAR expression. The PPAR forms heterodimers with Retinoid X receptor alpha to control microglial activation and phagocytosis (Heneka et al., 2005). PPAR inhibits pro-inflammatory cytokines that promote tau kinase hyperactivity, pTau buildup, and oxidative damage. Curcumin also promotes oligodendrocyte progenitor (OP) differentiation and inhibits tumor necrosis factor-induced OP maturation arrest through PPAR (Bernardo et al., 2021). Curcumin directly inhibits β-site amyloid precursor protein-cleaving enzyme 1 (BACE1), which catalyzes the N terminal cleavage of transmembrane amyloid precursor protein (APP) (Lin et al., 2008), which further indirectly inhibits BACE1 (Huang et al., 2020). Curcumin also reduce Aβ levels by delaying APP maturation in the secretory route (Zhang et al., 2010). The anti-inflammatory properties of curcumin have been linked to improved learning and memory in ApoE4 mice (Kou et al., 2021).

Curcumin combats AD in various ways, according to recent research (Serafini et al., 2017; Su et al., 2020). It inhibits the production of β-amyloid, tau, and acetylcholinesterase, controls microglia, and chelates metals (Tang et al., 2017; Voulgaropoulou et al., 2019). Curcumin binds to Aβ and inhibits harmful aggregate formation (Ono et al., 2004; Kim et al., 2005; Yang et al., 2005). However, the diketone bridge in curcumin is not necessary for curcumin’s anti-inflammatory actions, since reduced curcumin (tetrahydro curcumin) has strong anti-inflammatory characteristics (Begum et al., 2008). By deactivating Glycogen synthase kinase 3 (GSK-3), it reduced Aβ generation and plaque formation by downregulating the ROS/JNK pathway. Curcumins inhibition of BACE1 by GSK3 resulted in reduction of Aβ plaques (Durairajan et al., 2012). The precise means by which curcumin regulates these processes are unknown. It seems like that curcumin is a potential antioxidant for treating AD, and that combining carriers and targeting agents to enhance brain delivery is highly effective. In future, more research on curcumin’s mechanism of action related to NDs are required.

## Interplay of Curcumin and Unfolded Protein Response

The ER has vital cellular functions including protein folding, post-translational modification, protein translocation, lipid synthesis, and Ca^2+^ storage (Schwarz and Blower, 2016). Under ER-stress, evolutionarily conserved UPR response system corrects ER homeostasis by activating transcription factors (ATF4, ATF6, and XBP1) that inhibit protein translation, and promotes unfolded protein destruction (Zhao and Ackerman, 2006; Limonta et al., 2019). In cases of persistent ER stress, the UPR initiates intrinsic apoptotic pathway and cell death (Sano and Reed, 2013). UPR may also be triggered by non-ER stress associated mechanisms (Hetz et al., 2020). For example, vascular endothelial growth factor signaling promotes angiogenesis through the UPR pathway (Urra and Hetz, 2014). Considering the vastness of this topic, we have chosen to concentrate on curcumin’s role in UPR regulation in NDs.

### Curcumin as a Suppressor or Inducer of Unfolded Protein Response in Neurological Diseases

#### Curcumin Function in Brain Injury

Diffuse axonal injury (DAI) associated with abnormally expressed β-APP and p-tau proteins in neurons leds to ER-stress induced cell death. Curcumin treatment in rat DAI model increased PERK phosphorylation and decreased CHOP expression and therefore prevented aberrant protein accumulation and inhibited UPR pathway activation (Huang et al., 2018). In another study, curcumin protected against glutamate-induced hippocampus neurotoxicity. The therapeutic role of curcumin against various human diseases are well explored (Shakeri et al., 2019).

#### Mutation Associated Neuropathies

Besides having a protective impact on brain injuries, curcumin treatment has also improved peripheral neuropathies. For example, Trembler-J is caused by accumulation of mutated myelin proteins (PMP22) that led to ER stress, UPR activation, and Schwann cell death, which were minimized by curcumin treatment (Okamoto et al., 2013). The second most prevalent autosomal dominant hereditary demyelinating neuropathy is Charcot–Marie–Tooth disease type 1B (CMT1B), caused by activation of UPR components coupled with accumulation of mutant protein myelin protein Zero (P0, MPZ), as a consequence of ER stress (Santoro et al., 2004; Khajavi et al., 2005; Saporta et al., 2011). Using the CMT1B mouse model of human neuropathy, researchers discovered that these mice exhibited motor impairment and axonal abnormalities linked with aberrant UPR activation (Patzkó et al., 2012). It was noted that curcumin formulation could influence the treatment outcomes. Oral curcumin in sesame oil enhanced neurophysiological state and Schwann cell myelination in CMT1B mouse model with decreased UPR signaling (Patzkó et al., 2012). Using the HT22 mouse hippocampus cell line, Chhunchha et al. (2013) discovered that curcumin has anti-oxidative and anti-ER stress properties. Curcumin therapy increased peroxiredoxin 6 (Prdx6) expressions and decreased ER stress in hypoxic HT22 cells (Chhunchha et al., 2013). ApoE4 is the major genetic risk factor for AD associated with dementia. Kou et al. (2021) found that ApoE4 transgenic mice had impaired cognitive capacity, which is linked to ER stress and activation of inflammatory signaling in the nervous system; these were reversed by curcumin treatment in AD mice. Curcumin is also found effective in Pelizaeus-Merzbacher disease of mice model (Gow et al., 1998; Hübner et al., 2005; Yu et al., 2012).

### Analogs of Curcumin and Unfolded Protein Response in Neurological Diseases

The low bioavailability of curcumin leads to its poor absorption, requiring large doses of curcumin to reach a definite level in plasma. Curcumin plasma levels have been improved by dissolving it in various solutions, coating it with nanoparticles, forming emulsions and by creating its analogs (Sasaki et al., 2011; Zhongfa et al., 2012; Ramalingam and Ko, 2015). There are multiple curcumin analogs have been generated those presented profound effect in modulating ER stress in various cancers model including ovarian, colon, lung, prostrate, gastric, acute promyelocytic leukemia, glioblastoma, melanoma, and triple negative breast cancer cells (Zhang et al., 2012; Qu et al., 2013; Tan et al., 2014; Yoon et al., 2014; Zheng et al., 2014; Chen et al., 2016; Gao et al., 2017; He et al., 2021). However, curcumin mimics’ effects on neurodegenerative disorders are poorly documented.

Treatment with CNB-001, a curcumin derivative, reduced intracellular soluble-amyloid build up in AD transgenic mice by activating the UPR’s eIF2/ATF4 signaling (Valera et al., 2013). Protein disulfide isomerase (PDI) is an ER-resident chaperone that is modified to S-nitroso-PDI in the presence of high levels of nitric oxide (NO), which disrupts PDI’s redox activity and resulted in the accumulation of misfolded in AD and PD model (Uehara et al., 2006). Curcumin analog 3,5-bis (2-flurobenzylidene) piperidin-4-one (EF-24) pretreatment of neuroblastoma cell line SHSY-5Y cells prevented rotenone-induced (a mitochondrial reactive oxygen species elevator) reduction in PDI expression and ER stress associated protein aggregation (Pal et al., 2011). Glioblastoma is the most common, highly invasive and malignant form of brain cancer currently treated with surgery, radiotherapy and chemotherapy. In a study published by Sansalone et al. (2019) have generated 19 curcumin analogs, out of which 4 have induced glioma stem cells (GSC) death and prevented neurosphere formation. Mechanistically, curcumin analog robustly induced UPR signaling as detected by increased expression of CHOP, p-jun and caspase 12 markers (Sansalone et al., 2019). Another study by Hackler et al. (2016) has demonstrated the cytotoxic effect of curcumin derivative C-150 (Mannich-type) on eight glioma cell lines. C-150 treatment in gliomas cells significantly affected expression of UPR proteins, Akt, and PKCα activity.

Overall, curcumin and its derivatives are neuroprotective in various neurological disorders and kill cancer cells via modulating UPR signaling (Table 1). The main difficulty is to formulate curcumin or its counterpart in the correct dosage and administer it in a proper manner. This includes undoubtedly to overcome poor absorption, rapid metabolism and poor bioavailability of curcumin and substantially improve its beneficial activities.

**TABLE 1:** Curcumin and its analogs are associated with UPR modulation in neurological disorders.

Compound	Disease type/cell line	Effects on ER stress markers and UPR signaling	References
Curcumin	DAI model of rat	↑ (Nrf2, p-PERK), ↓ (CHOP)	Huang et al., 2018
Curcumin	Hippocampus or SH-SY5Y cells	↑ (AMPK), ↓ (p-IRE1α, p-PERK, NLRP3, TXNIP/NLRP3)	Li et al., 2015
Curcumin	Mouse hippocampus cell HT22	↑ Prdx6, CHOP, Grp78), ↓ (ROS)	Valera et al., 2013
Curcumin	Pmp22 Trembler-J mice	↓ (Atf3 and Ero1-lβ)	Okamoto et al., 2013
Curcumin dissolved in sesame oil or phosphatidylcholine	MPZ*^R98C^* knock-in mice	↓ (Bip, ATF6, spliced XBP1), no change in CHOP expression	Patzkó et al., 2012
Curcumin	ApoE4 transgenic mice (SCXK2016-0004)	↓ (NFkβ, APoE4, Grp78, IRE1α)	Kou et al., 2021
Curcumin	Transgenic myelin synthesis deficient model	No changes in expression of Grp78, CHOP, Gadd45a, calnexin, calreticulin, Herpud1	Yu et al., 2012
CNB-001 (Curcumin analog)	huAPPswe/PS1E9 transgenic mice and MC65 cells	↑ (p-PERK, eIF2α, HSP90, ATF4), ↓ (5-LOX, β-amyloid)	Valera et al., 2013
EF-24 (Curcumin analog)	SHSY-5Y	↑ (PDI expression), ↓ (AD associated protein aggregation)	Pal et al., 2011
Bis-chalcone 4j (Curcumin analog)	GSC lines Glio3, Glio4, Glio9, Glio11 and Glio14	↑ (CHOP, p-jun and caspase 12)	Sansalone et al., 2019
C-150 (Curcumin analog)	GBM1-6, U87 MG, U251 MG and U373 MG	↑ (Grp78, GADD153, ATF4, XBP1), ↓ (NFkβ, Akt, PKCα kinase activity)	Hackler et al., 2016

*DAI, diffuse axonal injury; Nrf2, nuclear factor erythroid-derived 2-like 2; CHOP, CCAAT-enhancer-binding protein homologous protein; AMPK, AMP-activated protein kinase; NLRP3, NLR family pyrin domain containing 3; ApoE4, Apolipoprotein E4; GSC, glioma stem cells; AD, Alzheimer’s disease; XBP1, X-binding protein-1; ATF, activating transcription factor; Grp78, 78-kDa glucose-regulated protein; PDI, protein disulfide-isomerase; HSP90, heat shock protein 90; GADD153, growth arrest- and DNA damage-inducible gene 153; PKCα, protein kinase C; eIF2α, eukaryotic translation initiation factor 2A; Bip, binding immunoglobulin protein; Ero1, endoplasmic oxidoreductin-1; Prdx6, peroxiredoxin-6; ROS, reactive oxygen species.*

## Curcumin, Unfolded Protein Response and Neurodegenerative Diseases: A Trivial Connection and Future Perspectives

The studies suggest that deregulation of proteostasis, particularly aberrant UPR signaling, may be a common pathogenic mechanism in the development of ND. While modulation of the UPR in animal illness models, including AD, has shown early promises. To determine whether UPR signaling is a protective mechanism or actively contributes to disease development, neuropathological data alone cannot be used. The idea of targeting the UPR, and specifically the PERK signaling is extremely interesting (Das et al., 2015). Some protein misfolding in NDs appear to benefit from selectively increasing protein synthesis upstream or downstream of eIF2a-P to avoid pancreatic toxicity associated with systemic PERK inhibition.

Researchers also discovering novel UPR activation methods, e.g., mitochondria-associated ER membranes are gaining popularity as a possible therapeutic target in NDs. Disrupted connections between the ER and mitochondria have been identified were curcumin seems to influence ER-mitochondrial interactions (Paillusson et al., 2016). Curcumin as licensed drug delaying the development of dementia in different model systems, this is an intriguing idea and a major step forward in the quest for a therapeutic agent for neurodegeneration. The next issue will be identifying how best and when to regulate the PERK pathway in patients, given that there are many proven therapeutic targets along the route. What remains unclear if curcumin may directly interfere in neurodegeneration without engaging UPR components?

Curcumin affect the action of many factors such as NF-κ B and AP-1 (Han et al., 2002). Curcumin binds to different proteins and enzymes and modulate their conformation and biological activities. Curcumin’s linker length and flexibility make it ideal for binding to Aβ aggregates. Curcumin’s unique structure, which consists of an, α, β-unsaturated β-diketone moiety linked by a seven carbon heptadiene chain, allows it to remain in keto–enol tautomeric forms in solution depending on the pH. When the pH is between 3 and 7, it is in the keto form, whereas the enol form is found around pH 8. Curcumin retains its coplanarity and extends the double-bond conjugation through six membered hydrogen bonding at the center when it is in the enol form which has strong Aβ aggregate binding activities. Curcumin, on the other hand, has very low binding activities for Aβ aggregates when it is in the keto form. Studies have shown that to be able to bind to Aβ aggregates, compounds need to be coplanar and have a double-bond conjugation of certain length. Curcumin, however, has certain disadvantages too, which includes its low water solubility and bioavailability. Dissolving it into organic solvent improves it solubility but its absorption remains poor (Ege, 2021; Jia et al., 2021). Some recent articles addressed the methods to enhance curcumin’s effectiveness in treating AD (Fan et al., 2018; Francesco et al., 2019; Ege, 2021). In future curcumin compounds must be chemically screened on target enzymes and proteins to facilitate more information.

Overall, the studies suggest that curcumin may prevents or postpones the onset of NDs by decreasing ER stress which seems to be responsible for NDs through a complicated processes (Figure 1). Additional mechanistic studies are needed to establish curcumin’s role in reducing ER stress. Despite promising preclinical findings, there are currently no clinical data to support curcumin as part of a drug therapy against NDs. The recent rise in the number of NDs patients across the globe suggests that researchers and practitioners will need to discover an effective pharmaceutical medication or therapy to successfully treat these illnesses in the future. Curcumin’s interaction and mechanism of action against NDs warrants a more research to accomplish this goal.

## Author Contributions

NJ, RP, AP, and SK contributed to the conception and design of the study. SM, AM, GP, NJ, and AP organized the material and wrote the first draft of the manuscript. SM, GP, SB, SH, NJ, and AP wrote sections of the manuscript. RP, VR, NJ, AP, and SK helped with manuscript editing and formatting. All authors contributed to manuscript revision, read, and approved the submitted version.

## Conflict of Interest

The authors declare that the research was conducted in the absence of any commercial or financial relationships that could be construed as a potential conflict of interest.

## Publisher’s Note

All claims expressed in this article are solely those of the authors and do not necessarily represent those of their affiliated organizations, or those of the publisher, the editors and the reviewers. Any product that may be evaluated in this article, or claim that may be made by its manufacturer, is not guaranteed or endorsed by the publisher.
